# Lysinoalanine cross-linking is a conserved post-translational modification in the spirochete flagellar hook

**DOI:** 10.1093/pnasnexus/pgad349

**Published:** 2023-10-26

**Authors:** Michael J Lynch, Maithili Deshpande, Kurni Kurniyati, Kai Zhang, Milinda James, Michael Miller, Sheng Zhang, Felipe J Passalia, Elsio A Wunder, Nyles W Charon, Chunhao Li, Brian R Crane

**Affiliations:** Department of Chemistry and Chemical Biology, Cornell University, Ithaca, NY 14853, USA; Department of Chemistry and Chemical Biology, Cornell University, Ithaca, NY 14853, USA; Philips Institute for Oral Health Research, Virginia Commonwealth University School of Dentistry, Richmond, VA 23298, USA; Philips Institute for Oral Health Research, Virginia Commonwealth University School of Dentistry, Richmond, VA 23298, USA; Department of Microbiology, Immunology, and Cell Biology, Robert C. Byrd Health Sciences Center, West Virginia University, Morgantown, WV 26505, USA; Department of Biochemistry, Robert C. Byrd Health Sciences Center, West Virginia University, Morgantown, WV 26505, USA; Proteomics and Metabolomics Facility, Institute of Biotechnology, Cornell University, Ithaca, NY 14853, USA; Department of Epidemiology of Microbial Diseases, Yale School of Public Health, New Haven, CT 06510, USA; Department of Epidemiology of Microbial Diseases, Yale School of Public Health, New Haven, CT 06510, USA; Department of Pathobiology and Veterinary Science, University of Connecticut, Storrs, CT 06269, USA; Department of Microbiology, Immunology, and Cell Biology, Robert C. Byrd Health Sciences Center, West Virginia University, Morgantown, WV 26505, USA; Philips Institute for Oral Health Research, Virginia Commonwealth University School of Dentistry, Richmond, VA 23298, USA; Department of Chemistry and Chemical Biology, Cornell University, Ithaca, NY 14853, USA

**Keywords:** spirochetes, bacterial flagella, FlgE, lysinoalanine, posttranslational modification

## Abstract

Spirochetes cause Lyme disease, leptospirosis, syphilis, and several other human illnesses. Unlike other bacteria, spirochete flagella are enclosed within the periplasmic space where the filaments distort and push the cell body by the action of the flagellar motors. We previously demonstrated that the oral pathogen *Treponema denticola* (Td) and Lyme disease pathogen *Borreliella burgdorferi* (Bb) form covalent lysinoalanine (Lal) cross-links between conserved cysteine and lysine residues of the FlgE protein that composes the flagellar hook. In Td, Lal is unnecessary for hook assembly but is required for motility, presumably due to the stabilizing effect of the cross-link. Herein, we extend these findings to other, representative spirochete species across the phylum. We confirm the presence of Lal cross-linked peptides in recombinant and in vivo-derived samples from *Treponema* spp., *Borreliella* spp., *Brachyspira* spp., and *Leptospira* spp. As was observed with Td, a mutant strain of Bb unable to form the cross-link has greatly impaired motility. FlgE from *Leptospira* spp. does not conserve the Lal-forming cysteine residue which is instead substituted by serine. Nevertheless, *Leptospira interrogans* FlgE also forms Lal, with several different Lal isoforms being detected between Ser-179 and Lys-145, Lys-148, and Lys-166, thereby highlighting species or order-specific differences within the phylum. Our data reveal that the Lal cross-link is a conserved and necessary posttranslational modification across the spirochete phylum and may thus represent an effective target for the development of spirochete-specific antimicrobials.

**Significance Statement**

The phylum Spirochaetota contains bacterial pathogens responsible for a variety of diseases, including Lyme disease, syphilis, periodontal disease, and leptospirosis. The motility of these pathogens is a major virulence factor that contributes to infectivity and host colonization. The oral pathogen *Treponema denticola* produces a posttranslational modification (PTM) in the form of a lysinoalanine (Lal) cross-link between neighboring subunits of the flagellar hook protein FlgE. Herein, we demonstrate that representative spirochete species across the phylum all form Lal in their flagellar hooks. *Treponema denticola* and *Borreliella burgdorferi* cells incapable of forming the cross-link are nonmotile, thereby establishing the general role of the Lal PTM in the unusual type of flagellar motility evolved by spirochetes.

## Introduction

**Fig. 1.**
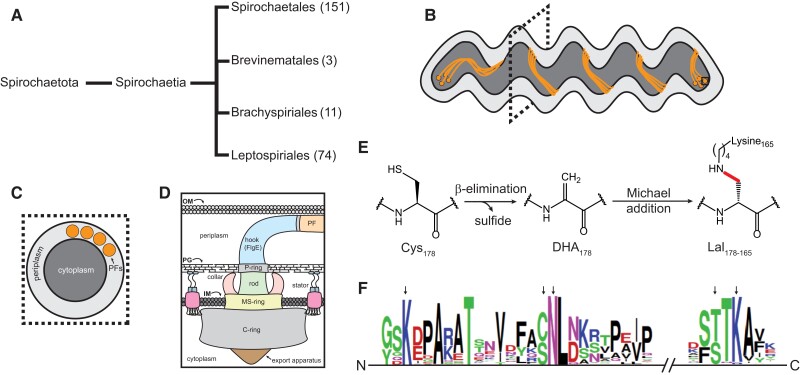
Lal cross-linking in the spirochete flagellar hook protein FlgE. A) Simplified taxonomic organization of the Spirochaetota phylum. The phylum is organized into a single class, which is further divided into four distinct orders. The approximate number of species present in each order is included for comparison (excluding environmental, unclassified, and uncultured species). B) Cartoon depiction of a spirochete cell with multiple subpolar membrane-embedded flagellar motors (solid black square) linked by a continuous ribbon of PFs. C) Cross-section of the spirochete cell body as the position indicated by the dotted square in B). The four PFs wrap around the cell body. D) Overview of the substructure of the bacterial flagellar motor (OM, outer membrane; PG, peptidoglycan; IM, inner membrane). E) Biochemical mechanism of Lal cross-link formation in the flagellar hook protein FlgE of Td. Residue numbering is based on the Td FlgE (Lal cross-link indicated by the thicker red line). F) MSA of the 241 FlgE sequences collected and filtered using AnnoTree (13). Black arrows (left to right, Td numbering) indicate the position of Lys-165, Cys-178, Asn-179, Thr-334, and Lys-336 residues. Sequence graphics created using WebLogo (14).

Pathogenic spirochetes cause several human and animal diseases, including Lyme disease, periodontal disease, syphilis, pinta, yaws, endemic syphilis, leptospirosis, swine dysentery, and bovine digital dermatitis (1–10). Currently, the phylum Spirochaetota is summarized as a single class, Spirochaetia, that is subdivided into four orders: Spirochaetales, Brevinematales, Brachyspirales, and Leptospirales (Fig. 1A) (11, 12). Although this classification scheme is relatively mature, as more spirochete species are identified, cultured, and their genomes sequenced, taxonomic organization continues to be updated for this complex collection of bacterial species (12, 15, 16).

Similar to other bacteria, spirochetes rely on chemotaxis to detect and migrate toward attractants (e.g. sugars, amino acids, rabbit serum, long-chain fatty acids, and hemoglobin) and away from repellants (e.g. ethanol and butanol) (17–22). Motility is a widely recognized virulence factor for pathogenic spirochetes (3, 7, 23–25). Similar to other flagellated bacteria, spirochete motility is powered by membrane-embedded rotary motors connected to helical flagellar filaments (Fig. 1B–D) (26). However, unlike the majority of flagellated bacteria which have external flagella like *Escherichia coli* and *Salmonella* spp., spirochetes enclose their flagellar filaments in the periplasm (7). These periplasmic filaments (PFs) wrap around the cell body and, in some species, form a continuous ribbon of overlapping PFs from pole to pole (Fig. 1B and C). To push the cell forward, PF rotation in the periplasm deforms the cell body, inducing undulations that travel as rolling waves along the cell body and producing thrust. Interestingly, in some species, PFs also serve a cytoskeletal role, warping the cell body to yield their distinct flat-wave or corkscrew morphology (1). PFs are connected to the motor via the hook (Fig. 1D). The hook is a highly curved, helically tubular structure comprised of ∼130 subunits of a single protein, FlgE (27). Within the motor, the hook serves to transmit motor torque to the PFs. As a result, the hook must be flexible enough to rotate within the periplasm yet strong enough to transfer torque to the filament-wrapped cell body and avoid buckling (28). FlgE monomers organize into 11 protofilaments that further assemble into a right-handed helix (29). To provide the flexibility required for rotation, extensive networks of protein–protein contacts between neighboring FlgE subunits strengthen the hook but also allow conformational change depending on the rotational state of the protofilament.

Although the structure of the bacterial flagella is generally well conserved across the bacterial kingdom, differences are common (26). In spirochetes, these include such examples as the addition a motor component known as the P-collar (30), glycosylation of the PFs (31), the use of multiple flagellin and sheath proteins in the PFs (32–35), and a lysinoalanine (Lal) posttranslational modification (PTM) found in FlgE (36–38). FlgE from *Treponema denticola* (Td) and *Borreliella burgdorferi* (Bb) self-catalyzes the formation of a Lal cross-link between conserved cysteine and lysine residues (Fig. 1E) harbored on adjacent FlgE subunits within the hook (36, 37). Unlike other forms of Lal found in nature, Lal formation in Td FlgE is autocatalytic and will occur in the absence of other enzymes, cofactors, or regulatory proteins (39). FlgE Lal cross-linking follows at least three distinct biochemical steps (Fig. 1E). First, Td FlgE oligomerizes via protein–protein interactions between long, highly conserved N- and C-terminal α-helical D0 domains (36). β-Elimination of the catalytic cysteine yields the reactive dehydroalanine (DHA) intermediate, producing hydrogen sulfide as a by-product (36, 40). Lastly, DHA reacts with the ε-NH2 group of lysine-165 in a Michael addition reaction to yield the mature Lal cross-link (red line in Fig. 1E). To date, we confirmed the presence of Lal cross-linked peptides in recombinantly derived Td FlgE and polyhook (PH) PFs from an engineered Bb strain lacking the hook length regulatory protein FliK (37). Mutagenesis of catalytic residues C178, K165, and structural residue N179 inhibited Lal formation in Td cells in vivo and rendered the cells nonmotile (37).

Although Td and Bb only represent one of the four orders that comprise the phylum Spirochaetota, multiple-sequence alignment data from spirochetes across the phylum suggest that Lal cross-linking is a conserved PTM (Fig. 1E). Herein, we test this hypothesis directly, detecting Lal cross-linking in in vitro*-* and in vivo*-*derived samples from *Treponema* spp., *Borreliella* spp., *Brachyspira* spp., and *Leptospira* spp. These species span genera and families and represent three out of the four orders in the phylum. Interestingly, our findings also reveal that *Leptospira interrogans* (Li) catalyzes multiple different Lal cross-links within the hook, a polymorphism likely conserved by other *Leptospira* spp. based on sequence conservation. Finally, swimming speed analysis of Bb cells harboring the C178A mutation in FlgE demonstrates the requirement of the Lal cross-linking for motility in this species as well as Td. Overall, these findings suggest that Lal cross-linking is a conserved PTM of the spirochete flagellar hook that is required for proper hook function and optimal motility.

## Results

### Td and Bb as model systems for identifying Lal cross-linking

**Fig. 2.**
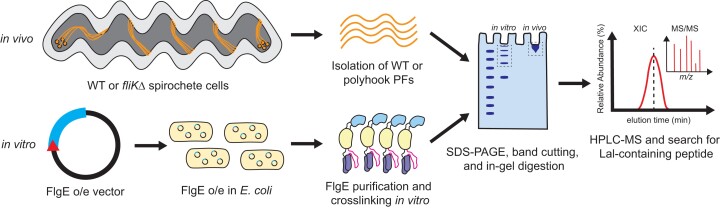
Overview of sample types used in this study. (Top) In vivo FlgE samples were derived from either WT or *fliK* knockout (*fliK*Δ) cell lines. (Bottom) In vitro samples were generated via recombinantly expressing spirochete FlgE proteins in *E. coli* cells and performing Lal cross-linking assays prior to MS analysis. All sample types were run on SDS-PAGE, and the appropriate HMW bands (dashed boxes) excised and submitted to MS for Lal detection.

For this study, we initially aimed to confirm the presence of Lal cross-linked FlgE peptides in unmodified spirochete strains. However, due to the challenging culturing conditions of many spirochete species, this was not feasible for certain spirochetes. As a result of these constraints, as well as the ability of FlgE proteins to self-catalyze the cross-link, we also explored recombinant samples when and/or if obtaining a wild-type (WT) PF sample was not possible (Fig. 2A). In vivo samples (Fig. 2A, top) originated from either WT PFs or from PH PFs. PH PFs were purified from engineered spirochete cells harboring a *fliK* gene deletion (*fliK*Δ) that removes the ability of cells to regulate their hook lengths. As a result, *fliK*Δ cells express elongated hooks that are several times longer than normal flagellar hooks (41). This approach was employed to increase the FlgE concentration compared with WT PF samples, thus increasing the probability of successfully identifying Lal cross-linked peptides via mass spectrometry (MS). If the WT or *fliK*Δ spirochete cells could not be cultured, the *flgE* gene was overexpressed in *E. coli* cells (Fig. 2, bottom). The proteins were then purified and incubated in cross-linking buffer to drive in vitro Lal cross-link formation (36, 37). Regardless of the source of FlgE protein, all sample types (WT, PH, and recombinant FlgE) were treated identically following in vivo PF isolation or in vitro Lal cross-linking assays. Previous studies have shown that endogenous FlgE proteins from Td and Bb form high-molecular-weight complexes (HMWCs) consisting of Lal cross-linked FlgE monomers when visualized via sodium dodecyl sulfate-polyacrylamide gel electrophoresis (SDS-PAGE) or western blots (37, 38). These HMWCs run at the top of SDS-PAGE gels, regardless of sample treatment prior to SDS-PAGE (37, 38). Therefore, recombinant or in vivo-derived FlgE samples were denatured and electrophoresed, and only the top region of the gel was extracted and submitted for high performance liquid chromatrography-mass spectrometry (HPLC-MS) analysis (dotted box in Fig. 2). To detect Lal-containing peptides by MS, we employed a similar procedure described previously for recombinant Td FlgE and PH Bb FlgE (37).

**Fig. 3.**
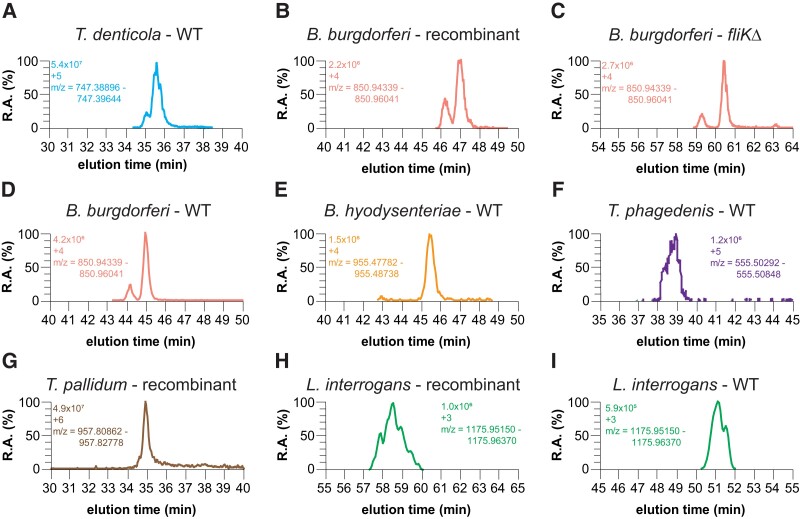
Detection of Lal cross-linked FlgE in recombinant, *fliK*Δ, and WT cell culture samples. XIC of A) Td WT PF, B) Bb recombinant FlgE, C) Bb *fliK*Δ, D) Bb WT PF, E) Bh WT PF, F) Tph WT PF, G) Tpa recombinant FlgE, H) Li recombinant FlgE, and I) Li WT PF Lal cross-linked FlgE peptides. For each XIC, the intensity levels, peptide charged state, and *m*/*z* range are shown.

To address whether Lal is a conserved PTM present in species throughout the phylum Spirochaetota, we initially examined Td and Bb FlgE (Fig. 3A–D) to demonstrate that recombinant FlgE cross-linking recapitulates the same Lal product as found in PH and/or WT samples. These two species belong to the order Spirochaetales but reside in different taxonomic families (Treponemataceae versus *Borreliella*, respectively). The Lal-containing tryptic peptides were confirmed in WT Td–derived PFs (Fig. 3A; Fig. S1). The extracted-ion chromatogram (XIC) and the electron-transfer dissociation (ETD) MS/MS fragmentation data agree with the expected mass and c/z fragmentation pattern of the parent Lal peptide. Taken together with our previous findings, we show that Lal cross-linked FlgE peptides are present in all three sample types tested (WT PFs, PH PFs, and recombinant Td FlgE). A similar approach with Bb FlgE samples produced similar results, with XIC and MS/MS data confirming the presence of Lal cross-linked peptides in both in vitro*-* and in vivo*-*derived Bb FlgE samples (Fig. 3B–D; Figs. S2–S4). To rule out a false-positive identification of Lal cross-linked peptides, we performed a similar experiment using Bb FlgE samples from a strain harboring a FlgE C178A mutation. No peptides of masses or charge states assigned to the Lal-containing peptides were detected in the XIC data from the C178A variant (Fig. S4D). Overall, in the test cases of Td and Bb, cross-linked peptides derived from three different FlgE sources related to the same strain all contain the same Lal species.

### Detection of FlgE Lal cross-links in diverse pathogenic spirochetes

We next sought to confirm the presence of Lal cross-linked FlgE peptides in other, more distantly related pathogenic spirochetes. We first examined the spirochete pathogen associated with swine dysentery, *Brachyspira hyodysenteriae* (Bh, formerly known as *Treponema* or *Serpulina*) (42). Bh belongs to the order Brachyspirales (Fig. 1A). Bh FlgE conserves lysine (149) and cysteine (162) residues analogous to those that form Lal (Lys165 and Cys178, respectively) in Td and Bb FlgE. Indeed, following cultivation of Bh and isolation of PFs, the XIC revealed peptides eluting at 61.08 min in the [M + 4H]^4+^ and [M + 5H]^5+^ charged states that matched the expected *m*/*z* of the Lal cross-linked Bh FlgE tryptic peptide (Fig. 3E; Fig. S5). The ETD MS/MS fragmentation of this peptide produced y and b ions with *m*/*z* values consistent with the expected Lal cross-linked peptide (Fig. S5).

In addition to the periodontal disease and gingivitis-associated pathogen Td, the genus *Treponema* has other notable pathogens that pose risks to humans and other mammals. These include the two *Treponema* species we examine here: *Treponema phagedenis* strain Kazan 5 (Tph) and *Treponema pallidum* subsp. *pallidum* strain Nichols (Tpa). Tph-like species are associated with bovine digital dermatitis (8). Inspection of the Tph FlgE primary sequence suggests that it also catalyzes Lal cross-links (Fig. S6A). Isolation and MS analysis of PH PFs from Tph WT cells yielded peptides in the [M + 4H]^4+^ and [M + 5H]^5+^ charged states that eluted between 37.6 and 37.7 min (Fig. 3F; Fig. S6). The ETD fragmentation pattern of the [M + 5H]^5+^ peptide produced c and z ions with *m*/*z* values consistent with the parent Lal cross-linked peptide, confirming the presence of Lal cross-linking in Tph FlgE (Fig. S6).

Tpa is one of four Tpa subspecies that are associated with human disease and is the pathogen responsible for venereal syphilis. The other three related strains are the causative agents of other human diseases like yaws (Tpa subsp. *pertenue*), endemic syphilis (Tpa subsp. *endemicum*), and pinta (*Treponema carateum*) (43). Formerly difficult to culture in vitro (43, 44), virulent Tpa cells were cultured both in vitro and isolated from rabbit testes in collaboration with Steve Norris and Diane Edmondson, University of Texas, McGovern Medical School, Houston, using their recently developed cell culture methodology (45–47). Unfortunately, the concentration of Tpa FlgE in the final WT PF samples was too low to confidently detect Tpa FlgE and any Lal cross-linked tryptic peptides. Given our previous data with Td and Bb FlgE, we produced Tpa FlgE recombinantly in *E. coli* and tested it for Lal cross-linking. In vitro cross-linking of Tpa FlgE yielded multimer HMW bands that formed in a time-dependent manner, albeit at a lower extent compared with similar concentrations of Td FlgE. Excision and in-gel digestion of the multimer bands yielded a peak on the MS XIC with an *m*/*z* range that corresponded to a Tpa FlgE Lal cross-linked peptide in a [M + 6H]^6+^ charged state (Fig. 3G). The Tpa FlgE Lal peptide was also detected in two other charged states ([M + 5H]^5+^ and [M + 7H]^7+^) that all eluted at ∼34.9 min (Fig. S7B). ETD MS/MS spectra of the [M + 6H]^6+^ peptide produced c and z ions that were consistent with the parent Lal cross-linked peptide (Fig. 3F; Fig. S7A and C).

**Fig. 4.**
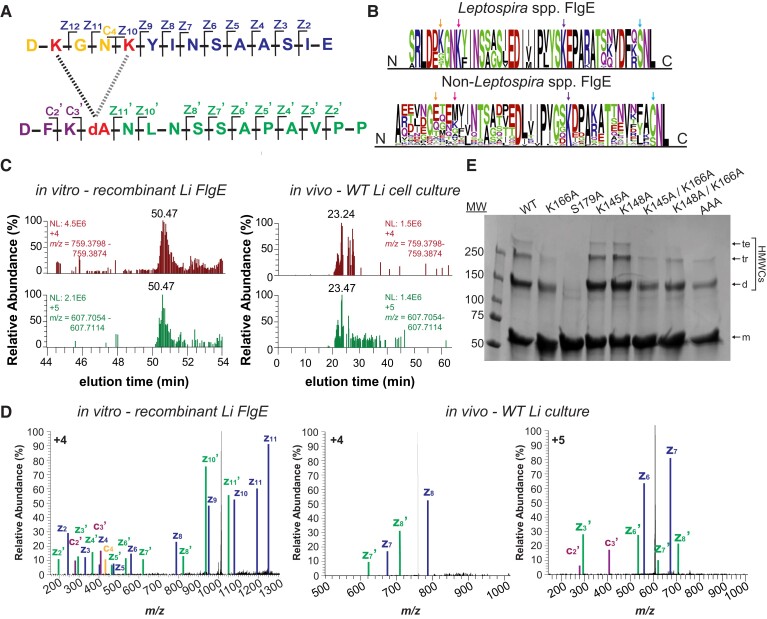
Li FlgE catalyzes multiple Lal cross-links. A) Li FlgE peptide derived from AspN digestion and cross-linked via Lal generated through the alternative sites of Lys-145, Lys-148, and DHA (dA)-179 (K, K and dA, red lettering). The two Lal cross-link isoforms are represented by a left, black (S179-K145) and right, gray (S179-K148) dotted line. c and z ions are indicated and color coded according to the peaks identified in D). B) MSA and sequence conservation of Lys-145 (leftmost orange arrow, Li numbering) and Lys-148 (second from left pink arrow) in *Leptospira* spp. FlgE (top) versus non-*Leptospira* spp. FlgE (bottom). Right-most purple and blue arrows mark the position of Lys-166 (K) and Ser-179 (S), respectively. C) XICs of the Lal cross-linked peptide shown in A) for recombinant in vitro FlgE polymerization (left) and for PFs derived from WT Li (right). D) MS/MS ETD fragmentation of Lal cross-linked peptide parent ion with c and z ions annotated and labeled according to A). On the left: MS/MS spectra of the Lal cross-linked peptide derived from in vitro polymerization of recombinantly produced FlgE ([M + 4H]^4+^). On the right: MS/MS spectra from FlgE derived from Li WT cells ([M + 4H]^4+^ and [M + 5H]^5+^, center and far right, respectively). Individual c and z ions were amplified 5–200×. E) In vitro Lal cross-linking assays of recombinant Li FlgE with lysine substitutions compared with FlgE with the WT sequence. SDS-PAGE analysis of cross-linked samples with arrows indicating recombinant Li FlgE monomer (m), dimer (d), trimer (tr), and tetramer (te) bands.

The order Leptospirales contains FlgE proteins that are the most diverse within the spirochetes with respect to Lal cross-linking. We examined Li serovar Copenhageni strain Fiocruz L1-130. Li belongs to the order Leptospirales (Fig. 1A) and is one of over 41 species and >300 serovars of pathogenic *Leptospira* responsible for the human and nonhuman disease known as leptospirosis (4, 48–51). Although several serogroups are associated with leptospirosis, serovar Copenhageni is recognized as one of the most virulent pathogenic species of *Leptospira* (4). Examination of the Li strain Fiocruz L1-130 FlgE primary sequence reveals that in addition to the conserved lysine residue, there is a notable difference in the Lal cross-link parent residues compared with the other spirochete species investigated previously. The conserved cysteine residue in non-*Leptospira* FlgE has been replaced with a serine residue in *Leptospira* spp. (Fig. S8A; Fig. 4A and B). Serine has been shown to participate in Lal formation in food processing (52). In addition, we have shown that Td FlgE C178S mutant can form Lal cross-linked HMWCs on SDS-PAGE gels in in vitro Lal cross-linking assays, and that Lal cross-linked peptides are present in these HMWCs via MS (36). Thus, we predicted that Li FlgE can also undergo Lal cross-linking. To test this possibility, we analyzed recombinant Li FlgE Lal cross-linked HMWCs produced in vitro and WT PFs purified from Li cells (Fig. 3H and I). XICs of both in vitro- and in vivo*-*derived Li FlgE samples showed peaks corresponding to Li FlgE Lal cross-linked peptides in the [M + 3H]^3+^ charged state (Fig. 3H and I; Figs. S8 and S9) and the [M + 4H]^4+^ and [M + 5H]^5+^ states (Figs. S8B and S9B). ETD fragmentation produced c and z ions that agreed well with the expected c and z ions of the AspN-digested Lal cross-linked peptide, therefore confirming the presence of Lal cross-linking in Li FlgE both in vitro and in vivo (Figs. S8C and S9C).

### Li FlgE produces several different Lal cross-links

In addition to the canonical Lal cross-link between K166 and S179 (Fig. 3H and I; Figs. S8 and S9) in Li FlgE, Lal also forms between S179 and either K145 and/or K148 (Fig. 4). These Lal isoforms were detected in both recombinantly expressed in vitro-derived Li FlgE and in vivo WT Li PFs (Fig. 4C; Figs. S8 and S9). Due to limited resolution of the MS and the proximity of K145 and K148 in sequence, it was unclear which residue was participating in the alternative cross-link. Sequence conservation of these residues in the order Leptospirales compared with species in the orders Brachyspirales, Brevinematales, and Spirochaetales (Fig. 4B) shows that K148 is conserved 100% of the time (67/67 sequences) compared with K145 being conserved 61% of the time (41/67 sequences), thereby suggesting that the Lal isoform more likely involves K148 compared with K145. A similar analysis with non-*Leptospira* spp. reveals that these two residues are highly variable, suggesting that Lal isoforms involving these positions are not common in other orders (Fig. 4B). To further verify these findings, we generated alanine substitutions for K145, K148, and K166 and measured the Lal cross-linking ability of these mutants compared with WT in the Lal cross-linking assay using recombinant Li FlgE (36). Compared with WT Li FlgE, the K166A substitution substantially reduced cross-linking, whereas the single substitutions K145A and K148A had no effect on the yield of HMWCs (Fig. 4E). Surprisingly, the K145A/K166A and K148A/K166A double Ala substitutions still formed cross-linked dimers and trimers, which suggested that either K145 or K148 could generate Lal isoforms. However, the triple substitution K145A/K148A/K166A did reduce the yield of cross-links compared with K166A combined with either K145A or K148A. Thus, K145 and K148 appear redundant in forming the alternative Lal linkage, and one appears to compensate for the absence of the other. Interestingly, the triple substitution K145A/K148A/K166A also produced some HMWCs, indicating that yet another lysine residue may have participated in the in vitro reaction with S179. Searching the WT PF MS data set for cross-links between S179 and other lysine residues did not yield any candidates other than K166, K145, and/or K148 (Fig. S10A). Furthermore, structural modeling of Li FlgE oligomers using cryo-electron microscopy density of *Salmonella* flagellar hooks showed no other lysine residues within reactive distance to S179 (Fig. S10B).

### Lal cross-linking is required for Bb motility

**Fig. 5.**
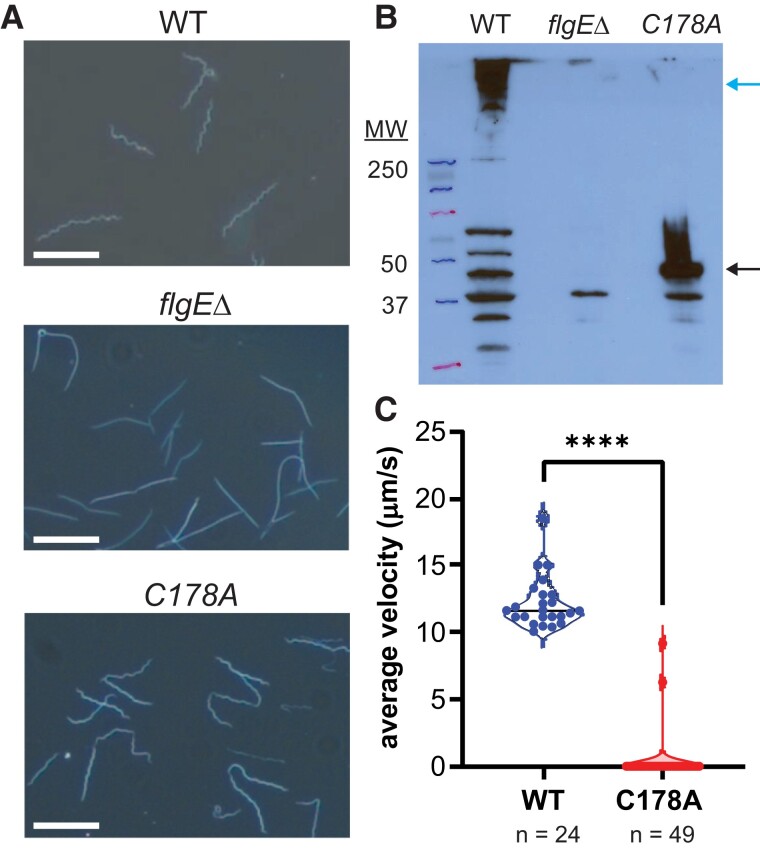
Motility of Bb depends on the presence of Lal cross-linked FlgE subunits. A) Cell morphology of WT, *flgE* knockout (*flgE*Δ), and FlgE C178A mutant cells (white scale bar = 10 µm). B) Detection of FlgE HMWCs (blue arrow) and monomers (black arrow) by immunoblotting with a specific antibody against Bb FlgE. C) Swimming speed analysis of individual Bb WT (*n* = 24) or FlgE C178A mutant (*n* = 49) cells. Significance was determined via a two-tailed Student's *t* test (****P < 0.0001). The figure was prepared using GraphPad Prism.

Previously, we showed that Lal cross-linking of FlgE is required for motility of the oral pathogen Td (38). To expand on this finding, we investigated whether Lal cross-linking was required for the motility of another genetically tractable pathogenic spirochete. We genetically modified low passage, virulent Bb strain B31 A3-68 cells to remove the *flgE* gene (*flgE*Δ) or expressed a C178A mutant form of the FlgE protein. In agreement with previous findings from Td, WT and C178A FlgE Bb cells had flat-wave morphology, whereas *flgE*Δ cells were rod shaped due to the lack of PFs (Fig. 5A). The WT morphology of Bb C178A FlgE mutant cells confirms the presence of correctly assembled hooks and PFs since PFs serve a cytoskeletal role in Td and Bb (53, 54). This observation suggests that hook and PF assembly does not depend on the presence of Lal cross-linking between adjacent FlgE subunits. Western blot analysis of whole-cell lysate of each strain confirmed the presence of FlgE HMWCs in WT Bb cells and the absence of FlgE in the *flgE*Δ mutant cells (Fig. 5B). Additionally, the western blot of the C178A FlgE strain showed only a FlgE band at the MW of the single subunit (Fig. 5B). Swimming speed analysis of WT (12 ± 2 µm/s, *n* = 24) and C178A (0.3 ± 2 µm/s, *n* = 49) cells reveals that despite having a normal morphology and correctly assembled PFs, the lack of Lal cross-links between adjacent FlgE monomers in the flagellar hook of Bb C178A cells renders the cells nonmotile.

## Discussion

Pathogenic spirochetes are responsible for several mammalian illnesses including Lyme disease, syphilis, periodontal disease, leptospirosis, pinta, yaws, endemic syphilis, swine dysentery, and bovine digital dermatitis. In these bacteria, motility is an essential virulence factor; loss of key flagellar genes attenuates infections for Bb, Bh, and Li (10, 25, 55–57). In addition to initial findings that FlgE cross-linking through Lal plays a critical role in the motility of Td (37), the Lal PTM appears to be found throughout spirochetes. Furthermore, the Lyme disease pathogen Bb also relies on Lal to produce functional periplasmic flagella. Using HPLC-MS coupled with ETD fragmentation of the parent Lal cross-linked peptide, Lal cross-linked FlgE peptides were detected from Td, Bb, Bh, Tph, Tpa, and Li. ETD-induced fragmentation was essential and superior to collision-induced dissociation (CID) for detecting and verifying the presence of Lal cross-linked peptides due to the tendency for Lal cross-linked peptides to assume highly charged states (e.g. +4, +5, and +6, Fig. S11). For Td and Bb, there is good agreement among in vitro- and in vivo-derived FlgE samples, with Lal cross-linked tryptic peptides being confidently detected in multiple charged states in all cases. These findings allowed us to analyze Lal cross-linking in clinically relevant spirochete pathogens that would otherwise not be easily obtained (e.g. Tpa). In total, Lal cross-linking was confirmed in a selection of spirochetes belonging to three out of the four Spirochaetota orders that represent the majority of the species (Fig. 1A). FlgE cross-linking was not analyzed in the smallest order, Brevinematales, which is comprised of only three genera (Brevinema, Thermospira, and Longinema). Each genus contains a single species (*Brevinema andersonii* [Ba], *Longinema margulisiae*, and *Thermospira aquatica*, respectively), when excluding environmental, unclassified, or uncultured species (11). FlgE sequences are not yet available for *L. margulisiae* and *T. aquatica*, but Ba FlgE maintains the conserved catalytic and/or regulatory residues required for Lal formation (Ba FlgE [accession ID: A0A1I1E638] K165, S178, and T328).

Li FlgE replaces the Lal precursor Cys residue with a Ser residue. Nonetheless, Li FlgE still self-catalyzes the Lal cross-link. In lantibiotic biosynthesis, serine and threonine residues are dehydrated to DHA and DHA and dehydrobutyrine, respectively (58). However, these transformations require the activation of the Ser/Thr Oγ via transesterification or phosphorylation (58). The ability to form Lal from recombinant FlgE in the absence of other factors indicates that similar activation reactions are not required per se; nevertheless, the Li in vitro reaction requires high pH (10–11). Due to the replacement of Cys with Ser in Li FlgE, the reactivity of Li FlgE is markedly lower compared with Td FlgE at similar concentrations. This observation agrees with previous data that showed lower HMWC production in in vitro Lal cross-linking assays of a Td FlgE C178S mutant compared with WT Td FlgE (36). Hence, the reduced reactivity of the serine substitution in Li FlgE may be compensated by other, currently unknown cellular factors. Unlike cross-linking in all other spirochetes tested, several different Lys residues can react with Li FlgE S179 to produce Lal. This lack of specificity would seem to not solely derive from the highly basic conditions of the in vitro reaction (pH 11), because alternative linkages are also found in the natural sample of Li PFs, and at least one of the alternative lysine residues is highly conserved in *Leptospira*.

Why *Leptospira* would catalyze different Lal cross-links in FlgE is currently unknown. We note that PFs from *Leptospira* spp. compared with non-*Leptospira* spp. may require enhanced flexibility that the heterogeneous cross-linking could impart. Torque measurements of Bb and Td motors yield values of ∼2,700 pN·nm and ∼800 pN·nm, respectively (59). In contrast, *Leptospira biflexa* motors generate a considerably higher torque of ∼4,000 pN·nm (59)—which compares with *E. coli* motors (∼4,500 pN·nm) (60). Furthermore, unlike Bb and Bh, which contain 14–22 and 16–18 PFs, respectively, and overlap in the center of the cell body, *Leptospira* spp. contain only two nonoverlapping PFs, one at each pole (1, 25). Whereas Td contains a comparable number of PFs (two PFs at each cell pole) as *Leptospira*, they overlap in the cell midbody, forming a ribbon of continuous PFs from pole to pole (31). Motility of *Leptospira* spp. is also more complex compared with *Borrelia* spp., *Brachyspira* spp., and *Treponema* spp. (7, 25). For non-*Leptospira* spp., asymmetric counter-clockwise/clockwise (CCW/CW) rotation of the PFs drives wave propagation of the cell body, thereby producing thrust to drive forward movement in the direction of the pole with motors undergoing CCW rotation (1, 7, 23). In contrast, *Leptospira* spp. form an asymmetric cell shape due to PF rotation with one end forming a left-handed helix and the other forming a hook shape (1, 7). Thrust is produced through the combined CCW rotation of the left-handed helical cell pole and CW rotation of the cell body, moving the cell in the direction of the helical pole (25, 61). One, if not all, of these factors may favor a flagellar hook in *Leptospira* with structural properties that are different from those of other spirochetes. Alternative Lal linkages will alter the constraints at the subunit interface owing to variable spacing and coupling to tertiary structure, perhaps allowing a tuning of hook flexibility to meet the unique demands of *Leptospira* motility.

In conclusion, FlgE cross-linking via Lal is an unusual PTM that appears to be an adaptation of spirochetes associated with their unusual cell structure and unique form of flagellar motility. The PTM is conserved across the order and, in at least Td and Bb, plays an essential role in motility, a key pathogenicity determinant in these bacteria. Such a modification does not exist in humans; thus, it represents a new target for development of antibiotics against pathogenic spirochetes.

## Materials and methods

### Sequence analysis of spirochete FlgE

Multiple sequence alignment (MSA) analysis was performed on FlgE sequences for spirochetes whose genomes have been sequenced and annotated. Sequences were retrieved using Annotree (13) with the following search parameters: Kyoto Encyclopedia of Genes and Genomes (KEGG): K02390; percent identity: 30; *E*-value: 0.00001; percent subject alignment: 70; and percent query alignment: 70. Sequences were obtained for all bacterial species whereas only spirochete sequences were analyzed. Individual spirochete FlgE sequences were filtered, and redundant sequences (both in sequence and species) were removed and aligned using Clustal Omega (62). WebLogo (14, 63) was used to create sequence conservation graphics for *Leptospira*, non-*Leptospira*, and all spirochete FlgE.

### Cloning, expression, purification, and Lal cross-linking of recombinant FlgE

For recombinant FlgE samples from Td, Bb, Li, and Tpa, the *flgE* gene was amplified from genomic DNA and inserted into a pet28a^+^ vector in-frame with an N-terminal His_6_-tag using Gibson assembly. All plasmids were confirmed by Sanger sequencing. For the expression, refolding, and purification of recombinant FlgE, the protocol described previously was followed without modification (36). Protein concentrations were determined using the BCA assay (Pierce, Cat. No. 23227). To form Lal cross-linked FlgE in vitro, 5–10 mg/mL recombinant FlgE was incubated with cross-linking buffer (50 mM Tris pH 8.5, 160 mM NaCl, and 1 M ammonium sulfate) for 2–5 days at 4°C. For *Leptospira* FlgE samples, cross-linking buffer was changed to 50 mM CAPS pH 11, 160 mM NaCl, and 1 M ammonium sulfate.

### Spirochete cell culture

Low-passage infectious clone A3-68 (WT), a derivative strain from Bb strain B31 A3, was used in this study (22). This strain was a kind gift from P. Rosa (Rocky Mountain Laboratories, NIAID, NIH). Cells were grown in liquid Barbour–Stoenner–Kelly II (BSKII) medium in the presence of 3.4% carbon dioxide with appropriate antibiotic(s) for selective pressure as needed, i.e. kanamycin (300 μg/mL) for *fliK*Δ mutant (64). Td ATCC 35405 strains (WT and Δ*fliK*) were grown anaerobically in oral bacterial growth medium containing 10% (v/v) heat-inactivated rabbit serum at 37°C as described previously (37). Bh WT B204 serotype 2 ATCC 31212 cells were cultured in a Coy chamber using a premixed gas mixture of ∼90% nitrogen and 10% CO_2_ at 37–38°C in brain heart infusion broth supplemented with 10% (v/v) fetal bovine serum (65). Tph Kazan 5 cells were grown anaerobically in peptone yeast extract glucose medium supplemented with 10% (v/v) heat-inactivated rabbit serum (66). Li serovar Copenhageni strain Fiocruz L1-130 cells were cultured in Ellinghausen–McCullough–Johnson–Harris (EMJH) liquid medium supplemented with 1% rabbit serum until they reached logarithmic phase at 30°C (67).

### PF purification

Isolation of spirochete PFs was performed as described previously, with modifications (31, 56, 68). Briefly, 500 mL–2 L of cells was grown as described above and harvested via centrifugation for 15 min at 8,000 × g. All centrifugation steps were performed at 4°C unless stated otherwise. Cell pellets were then resuspended in cold phosphate-buffered saline (PBS) pH 7.0, centrifuged again, resuspended in ∼6 mL of 150 mM Tris HCl pH 6.8 and 160 mM NaCl, and then centrifuged one final time. The cell pellet was then resuspended in ∼3 mL 150 mM Tris pH 6.8 and 160 mM NaCl and gently rocked for 5 min at 4°C. Resuspended cells were then treated with 300 µL 20% (v/v) Triton X-100, which was added slowly dropwise over 10 min, and stirred gently for 1 h at room temperature. Cell lysis was confirmed by dark-field microscopy, and the lysate clarified via centrifugation at 15,000 × g for 45 min. The crude pellet was then resuspended in 3 mL of 150 mM Tris HCl pH 6.8 and 160 mM NaCl, and 300 µL of 0.69 U/µL mutanolysin in 18.2 MΩ water was added and allowed to incubate at room temperature for 1 h and then overnight at 4°C. Samples were then centrifuged at 8,000 × g for 30 min, and the supernatant was collected. A saturated solution of ammonium sulfate was then added dropwise to the supernatant until a final concentration of 12.5% (m/v) was obtained and stirred for 20 min at 4°C. To pellet the WT and PH PFs, the supernatant was then centrifuged at 120,000 × g for 2 h and the pellet resuspended in 1 mL of 150 mM Tris HCl pH 6.8 and 160 mM NaCl. Samples were stored at 4°C for 1–2 days or flash frozen in liquid nitrogen and stored at −80°C.

### SDS-PAGE and in-gel digestion of Lal cross-linked FlgE

Cross-linked recombinant FlgE samples were directly mixed 1:1 with 2× SDS-PAGE loading dye supplemented with 100 mM dithiothreitol (DTT) and heated at 90°C for 10 min. Denatured samples were then centrifuged briefly and electrophoresed on a 4–20% denaturing Tris-glycine SDS-PAGE gel. The gel was stained with Coomassie blue, destained, and washed extensively with 18.2 MΩ water. Multimer HMW bands were then excised and submitted for MS. PF samples were prepared in a different manner. Briefly, PF samples were diluted 10× with acetone and incubated at −20°C for 20 min. Precipitated protein was then centrifuged briefly at 14,800 rpm, and the supernatant was discarded. The protein pellet was then briefly dried under a stream of nitrogen for 5 min to remove residual acetone, washed with cold 18.2 MΩ water, and resuspended in 100 µL 8 M urea. Samples were then electrophoresed according to the recombinant FlgE procedure. Excised HMW bands were sliced into ∼1-mm cubes and washed consecutively with 200 μL deionized water followed by 50 mM ammonium bicarbonate, 50% (v/v) acetonitrile, and finally 100% acetonitrile. The dehydrated gel pieces were reduced with 50 μL of 10 mM DTT in 100 mM ammonium bicarbonate for 1 h at 60°C, followed by alkylation with 50 μL of 55 mM iodoacetamide in 100 mM ammonium bicarbonate at room temperature in the dark for 45 min. Wash steps were repeated as described above. The gel was then dried and rehydrated with 100 μL trypsin and GluC (for Tph FlgE) or only AspN (for *Leptospira* FlgE) at 10 ng/µL in 50 mM ammonium bicarbonate and incubated on ice for 30 min and then at 37°C for 18 h. GluC and AspN were used for Tph and *Leptospira* FlgE samples, respectively, to reduce the size of the Lal cross-linked peptide for successful detection. Digestion was stopped by adding 30 µL 5% (v/v) formic acid. The digested peptides were extracted from the gel twice with 200 μL of 50% (v/v) acetonitrile containing 5% (v/v) formic acid and once with 200 μL of 75% (v/v) acetonitrile containing 5% (v/v) formic acid. Extractions from each sample were pooled together. The pooled sample was dried in SpeedVac SC110 (Thermo Savant, Milford, MA) to 200 μL to remove the acetonitrile (ACN) and then filtered with a 0.22-µm spin filter (Costar Spin-X from Corning), dried to dryness in the speed vacuum, and analyzed as trypsin, trypsin/GluC, or AspN digests. Each sample was reconstituted in 0.5% (v/v) formic acid prior to HPLC-MS/MS analysis.

### Identification of cross-links by nano-LC-MS/MS

The digested product was analyzed by nano-LC-MS/MS analysis at the Cornell Proteomics and Metabolomics Facility. The analysis was carried out using an Orbitrap Fusion Tribrid (Thermo Fisher Scientific, San Jose, CA) mass spectrometer equipped with a nanospray Flex Ion Source and coupled with a Dionex UltiMate 3000 RSLCnano system (Thermo, Sunnyvale, CA). Each sample was loaded onto a nano-Viper PepMap C18 trapping column (5 µm, 100 µm × 20 mm, 100 Å, Thermo Fisher Scientific) at 20 µL/min flow rate for rapid sample loading. After 3 min, the valve switched to allow peptides to be separated on an Acclaim PepMap C18 nanocolumn (2 µm, 75 µm × 25 cm, Thermo Fisher Scientific) at 35°C in either 60- or 90-min gradients of 5 to 35% buffer B (98% ACN with 0.1% formic acid) at 300 nL/min. The Orbitrap Fusion was operated in positive ion mode with nanospray voltage set at 1.85 kV and source temperature at 275°C. External calibrations for Fourier transform, ion trap, and quadrupole mass analyzers were performed prior to the analysis. Samples were analyzed using the CID and ETD toggle workflow, in which MS scan range was set to 350–1600 *m*/*z*, and the resolution was set to 120,000. Precursor ions with charge states 3–7 were selected for ETD MS/MS acquisitions in ion trap analyzer and an automatic gain control (AGC) target of 3 × 10 (4). The precursor isolation width was 3 *m*/*z*, and the maximum injection time was 118 ms. Precursor ions with charge states 2–3 were selected for CID MS/MS, and normalized collision energy was set to 30%. All data were acquired under Xcalibur 4.4 operation software and Orbitrap Fusion Tune application v3.5 (Thermo Fisher Scientific).

### MS data analysis

All MS and CID-ETD MS/MS raw spectra from each sample were searched using Proteome Discoverer 2.5 (Thermo Fisher Scientific, San Jose, CA) with the Sequest HT algorithm for identification of peptides. For each spirochete species (*Treponema* spp., *Borrelia* spp., *Brachyspira* spp., and *Leptospira* spp.), databases with targeted protein sequences were used for PD 2.5 database search. The search parameters were as follows: two missed cleavages for AspN or trypsin digestion with fixed carbamidomethyl modification of cysteine, variable modifications of methionine oxidation, asparagine, and glutamine deamidation, protein N-terminal Met-loss, acetylation, and Met-loss + acetylation. The peptide mass tolerance was 10 ppm, and MS/MS fragment mass tolerance was 0.6 Da. Only high confidence peptides defined by Sequest HT with a 1% false discovery rate (FDR) by percolator were considered for the peptide identification. Confident identification of Lal cross-linked peptides was achieved by manual confirmation of MS precursor ions with high mass accuracy and their associated CID and/or ETD MS/MS spectra.

### Site-directed mutagenesis of Bb

Site-directed mutagenesis was performed using Agilent QuikChange II site-directed mutagenesis kit (Agilent, Santa Clara, CA) according to the manufacturer's instruction. The plasmid that expresses FlgE recombinant protein was used as a template to replace Cys178 with alanine, using primers P1/P2 (see Table S1). All mutations were confirmed by DNA sequencing.

### Constructions of Bb FlgE deletion and site-directed mutants

The *flgE::kan* plasmid was constructed to replace the entire open reading frame of *flgE* with a kanamycin resistance cassette (*aphI*). To construct *flgE::kan*, the *flgE* upstream region, *aphI*, and the *flgE* downstream region were PCR amplified with primers P3/P4, P5/P6, and P7/P8, respectively (Table S1). The resultant PCR fragments were fused together with primers P3/P8. The fused PCR fragment was then cloned into the pGEM-T Easy vector (Promega, Madison, WI) generating *flgE::kan*. To delete *flgE*, *flgE::kan* was linearized and transformed into B31 A3-68 WT competent cells via electroporation as previously described (69). The deletion was confirmed by PCR and immunoblotting analyses. The resulting mutant was designated the *flgE*Δ strain. To construct *flgE* C178A, the full-length *flgE* C178A (flgE*) gene and the aadA1 cassette were PCR amplified with primers P9/P10 and P11/P12 (Table S1), respectively, and then fused together with primers P9/P12, generating flgE*-aadA1. The upstream and downstream region of flgE* was PCR amplified with primers P3/P13 and P14/P8 and then fused together with flgE*-aadA1 by PCR using primers P3/P8. The obtained DNA fragment was cloned into the pGEM-T Easy vector. To generate the C178A replacement of *flgE*, the flgE*-aadA1 vector was linearized and transformed into the *flgE*Δ mutant via electroporation (69). The resulting complemented clones were confirmed by PCR and immunoblotting analyses. Specifically, the primer set P9/P10 was used to amplify the flgE* gene and sequenced to confirm the flgE* replacement instead of the WT *flgE*. The primers for constructing these two mutants are listed in Table S1.

### Motion tracking analysis

The velocity of bacterial cells was measured using a computer-based bacterial tracking system, as previously described (17, 70). In brief, Bb WT and *flgE* mutant strains were first diluted (1:1) in BSKII medium, and then 20 μL of diluted cultures was mixed with an equal volume of 2% methylcellulose with a viscosity of 4,000 cP (MC4000). Then Bb cells were video captured with iMovie on an Apple Mac computer. Videos were exported as QuickTime movies and imported into OpenLab (Improvision Inc., Coventry, United Kingdom) where the frames were cropped, calibrated, and saved as LIFF files. Then the software package Volocity (Improvision Inc.) was used to track individual moving cells and calculate cell velocities. For each bacterial strain, at least 25 cells were recorded for up to 30 s.

### Structural modeling of *Leptospira* FlgE

To model the orientation of FlgE monomers in Li hooks, AlphaFold model of Li FlgE was obtained from the AlphaFold protein structure database (UniProt ID: A0A1N6RW72) (71). Li FlgE monomers were aligned to the D1–D2 domain segments of *Salmonella typhimurium* FlgE monomers as they are arranged in a fully assembled hook (PDB 6JZT) (72). Structural superimposition, analysis, and images were prepared in PyMol (73).

## Supplementary Material

pgad349_Supplementary_Data

## Data Availability

All mass spectrometry data have been deposited to the ProteomeXchange Consortium via the PRIDE partner repository with the data set identifier PXD045997. All other data are included in the article and/or supporting information.

## Preprint

This manuscript was posted as a preprint: https://doi.org/10.1101/2023.06.13.544825.
