# Supplementary material for: Lysinoalanine cross-linking is a conserved post-translational modification in the spirochete flagellar hook
Source: PNAS Nexus. 2023 Oct 26;2(12):pgad349. doi: 10.1093/pnasnexus/pgad349 (PMC10691653; doi:10.1093/pnasnexus/pgad349)
Supplement: pgad349_Supplementary_Data [file pgad349_supplementary_data.docx]

**Supplemental Figures and Tables**


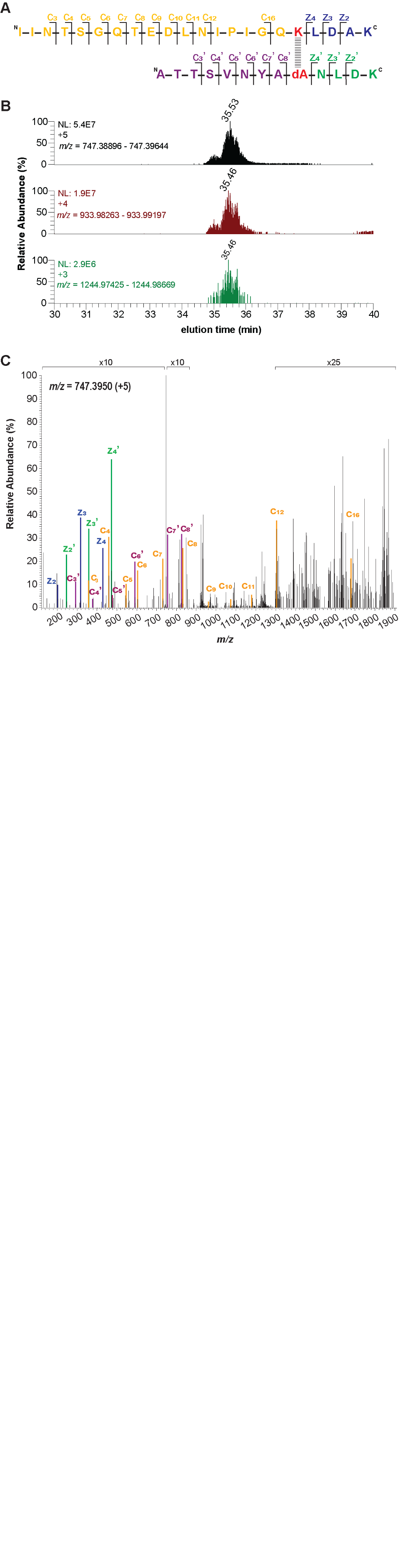


**Figure S1:** Lal-peptide detection in *T. denticola* WT PFs. (A) Trypsin-digested Lal-containing Td FlgE peptide with c and z ions labeled as shown in (C). Lysine-165 and DHA-178 (dA) are colored red and the Lal crosslink is represented by a dotted gray line. **(B)** XICs of the +3 (top), +4 (middle), and +5 (bottom) charged Lal crosslinked peptide shown in (A). **(C)** MS/MS ETD fragmentation spectrum of Lal-crosslinked peptide +5 parent ion with c and z ions annotated and labeled according to (A). Individual c and z ions were amplified 0-25x.


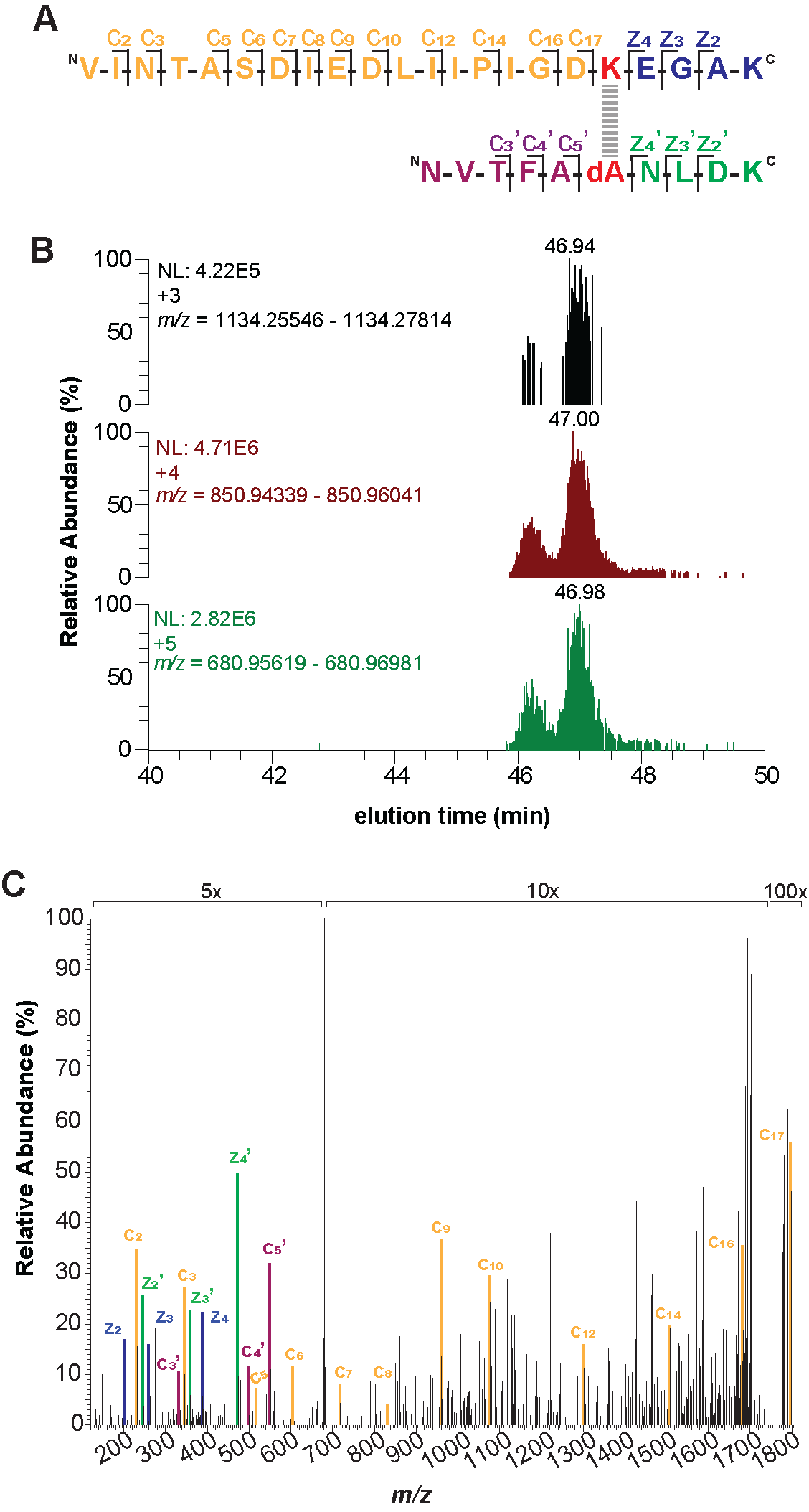


**Figure S2:** Lal-peptide detection in *B. burgdorferi* recombinant FlgE. (A) Trypsin-digested Lal-containing Bb FlgE peptide with c and z ions labeled as shown in (C). Lysine-165 and DHA-178 (dA) are colored red and the Lal crosslink is represented by a dotted gray line. **(B)** XICs of the +3 (top), +4 (middle), and +5 (bottom) charged Lal crosslinked peptide shown in (A). **(C)** MS/MS ETD fragmentation spectrum of Lal-crosslinked peptide +5 parent ion with c and z ions annotated and labeled according to (A). Individual c and z ions were amplified 5-100x.


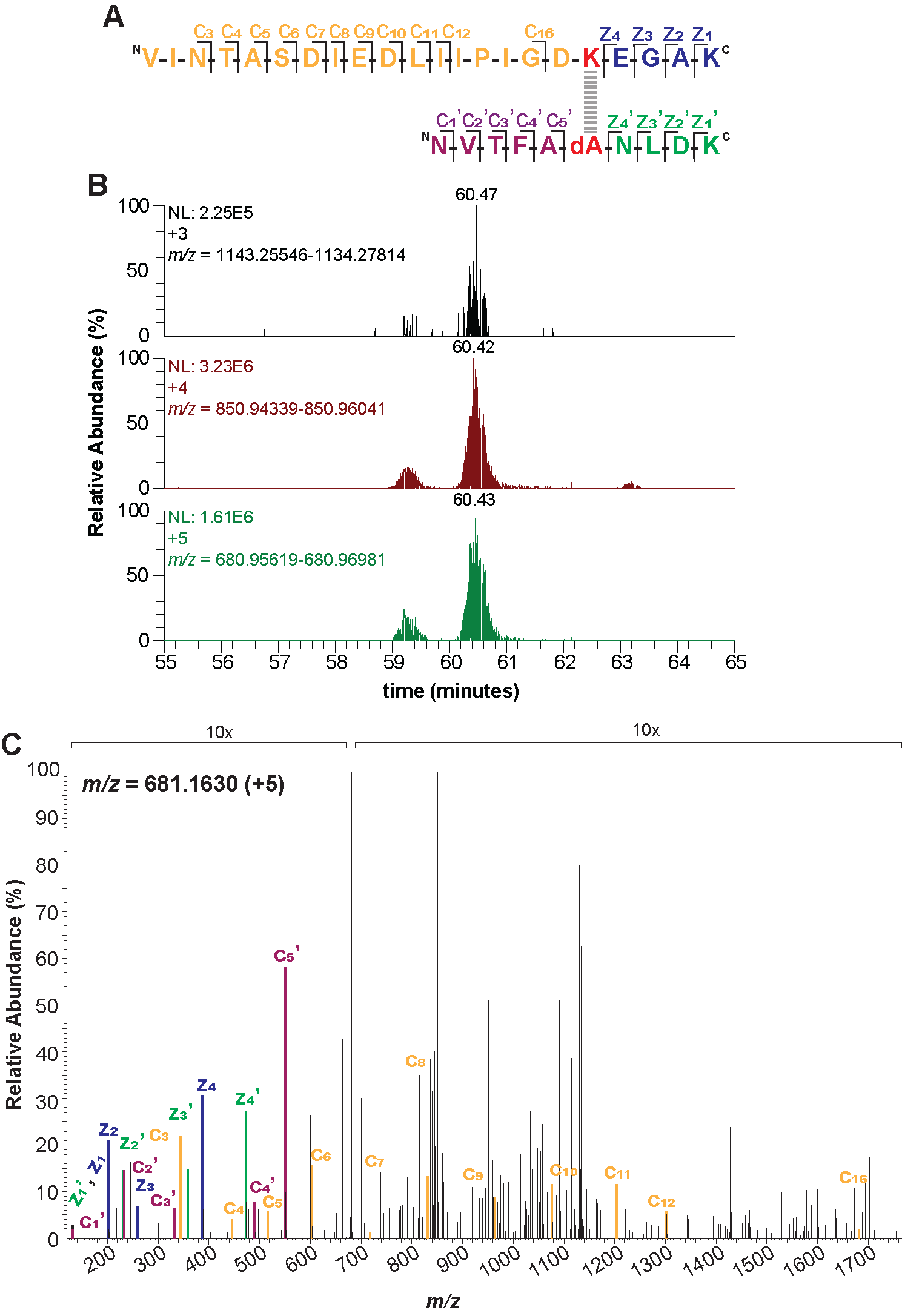


**Figure S3:** Lal-peptide detection in *B. burgdorferi fliK*Δ PH PFs. (A) Trypsin-digested Lal-containing Bb FlgE peptide with c and z ions labeled as shown in (C). Lysine-165 and DHA-178 (dA) are colored red and the Lal crosslink is represented by a dotted gray line. **(B)** XICs of the +3 (top), +4 (middle), and +5 (bottom) charged Lal crosslinked peptide shown in (A). **(C)** MS/MS ETD fragmentation spectrum of Lal-crosslinked peptide +5 parent ion with c and z ions annotated and labeled according to (A). Individual c and z ions were amplified 0-10x.


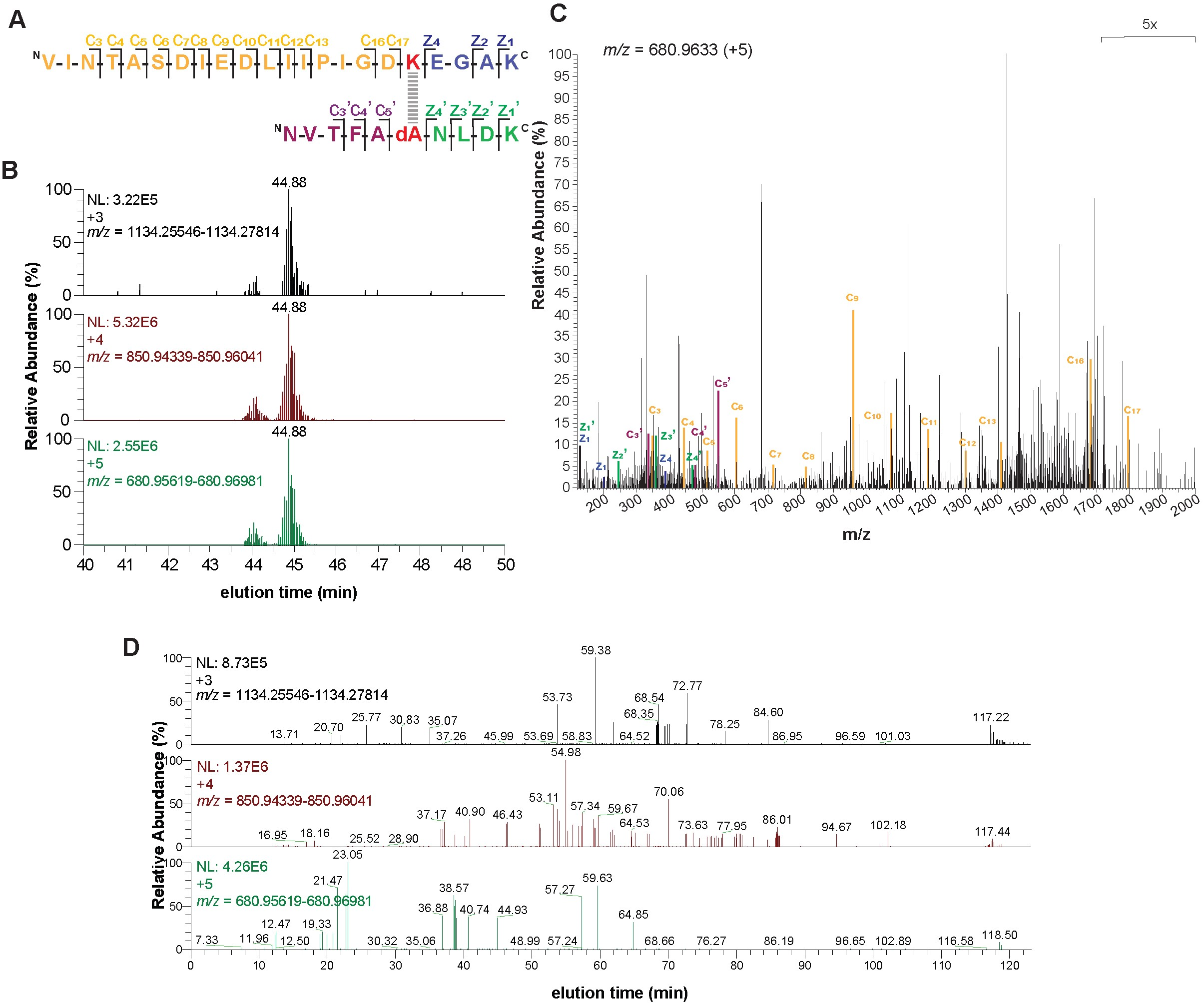


**Figure S4:** Lal-peptide detection in *B. burgdorferi* WT PFs. (A) Trypsin-digested Lal-containing Bb FlgE peptide with c and z ions labeled as shown in (C). Lysine-165 and DHA-178 (dA) are colored red and the Lal crosslink is represented by a dotted gray line. **(B)** XICs of the +3 (top), +4 (middle), and +5 (bottom) charged Lal crosslinked peptide shown in (A). **(C)** MS/MS ETD fragmentation spectrum of Lal-crosslinked peptide +5 parent ion with c and z ions annotated and labeled according to (A). Individual c and z ions were amplified 0-5x. **(D)** XICs of the +3 (top), +4 (middle), and +5 (bottom) from Bb C178A FlgE mutant cells.


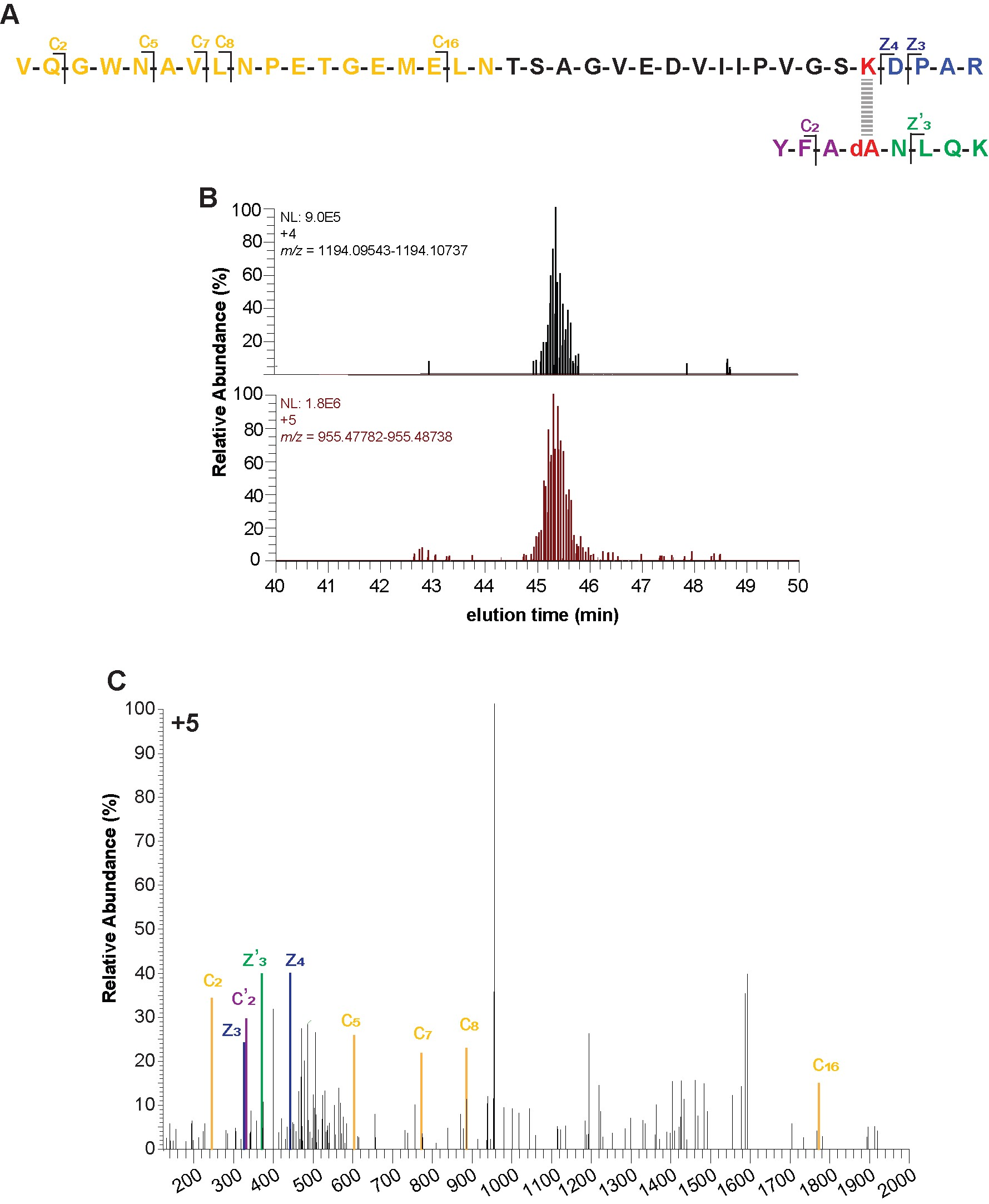


**Figure S5:** Lal-peptide detection in *B. hyodysenteriae* WT PFs. (A) Trypsin-digested Lal-containing Bh FlgE peptide with y and b ions labeled as shown in (C). Lysine-149 and DHA-162 (dA) are colored red and the Lal crosslink is represented by a dotted gray line. **(B)** XICs of the +4 (top) and +5 (bottom) charged Lal crosslinked peptide shown in (A). **(C)** MS/MS ETD fragmentation spectrum of Lal-crosslinked peptide +4 parent ion with y and b ions annotated and labeled according to (A). Individual y and b ions were amplified 0-50x.


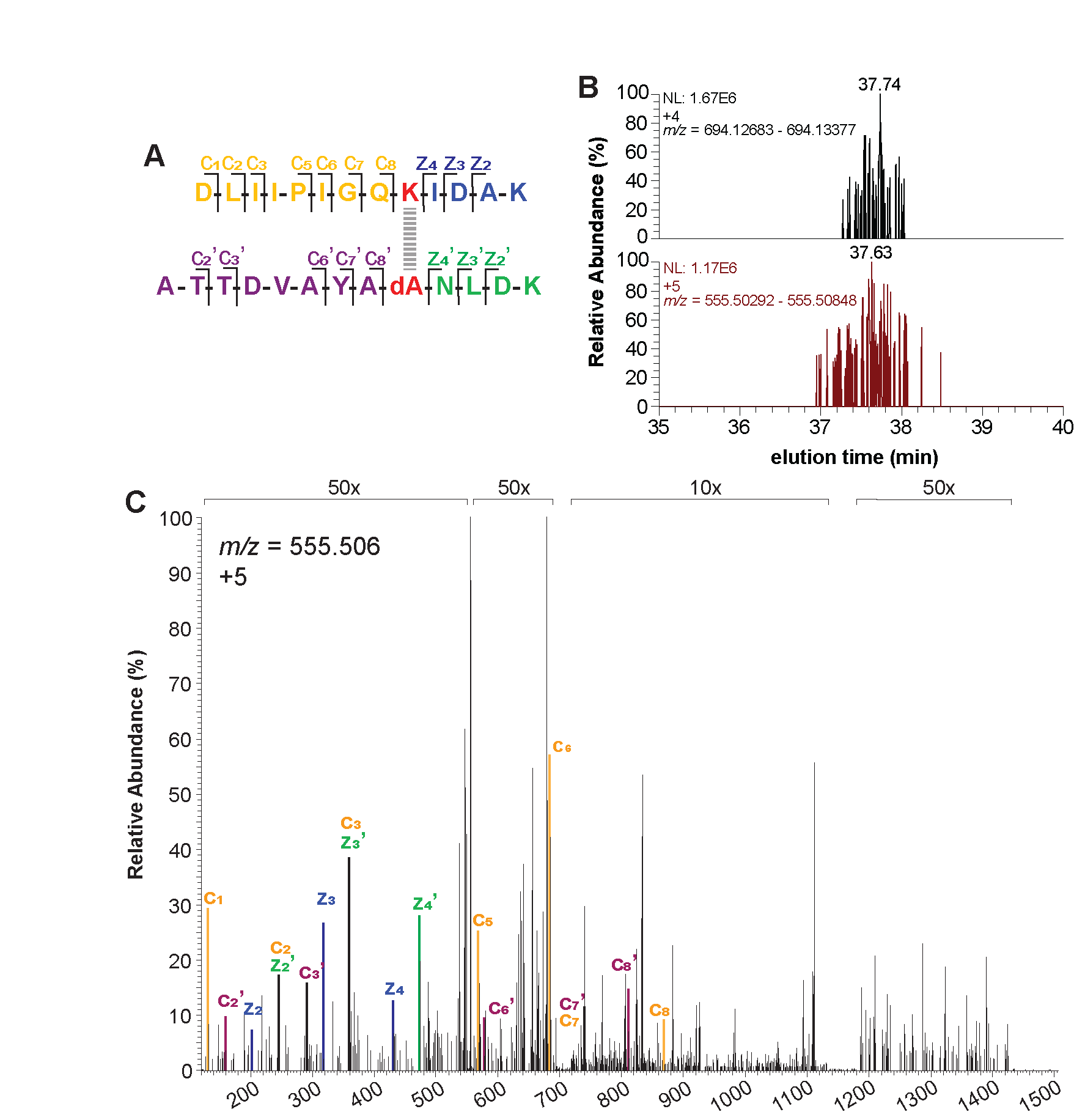


**Figure S6:** Lal-peptide detection in *T. phagedenis flik*Δ PH PFs. (A) Trypsin-digested Lal-containing Tph FlgE peptide with c and z ions labeled as shown in (C). Lysine-165 and DHA-178 (dA) are colored red and the Lal crosslink is represented by a dotted gray line. **(B)** XICs of the +4 (top) and +5 (bottom) charged Lal crosslinked peptide shown in (A). **(C)** MS/MS ETD fragmentation spectrum of Lal-crosslinked peptide +5 parent ion with y and b ions annotated and labeled according to (A). Individual c and z ions were amplified 10-50x.


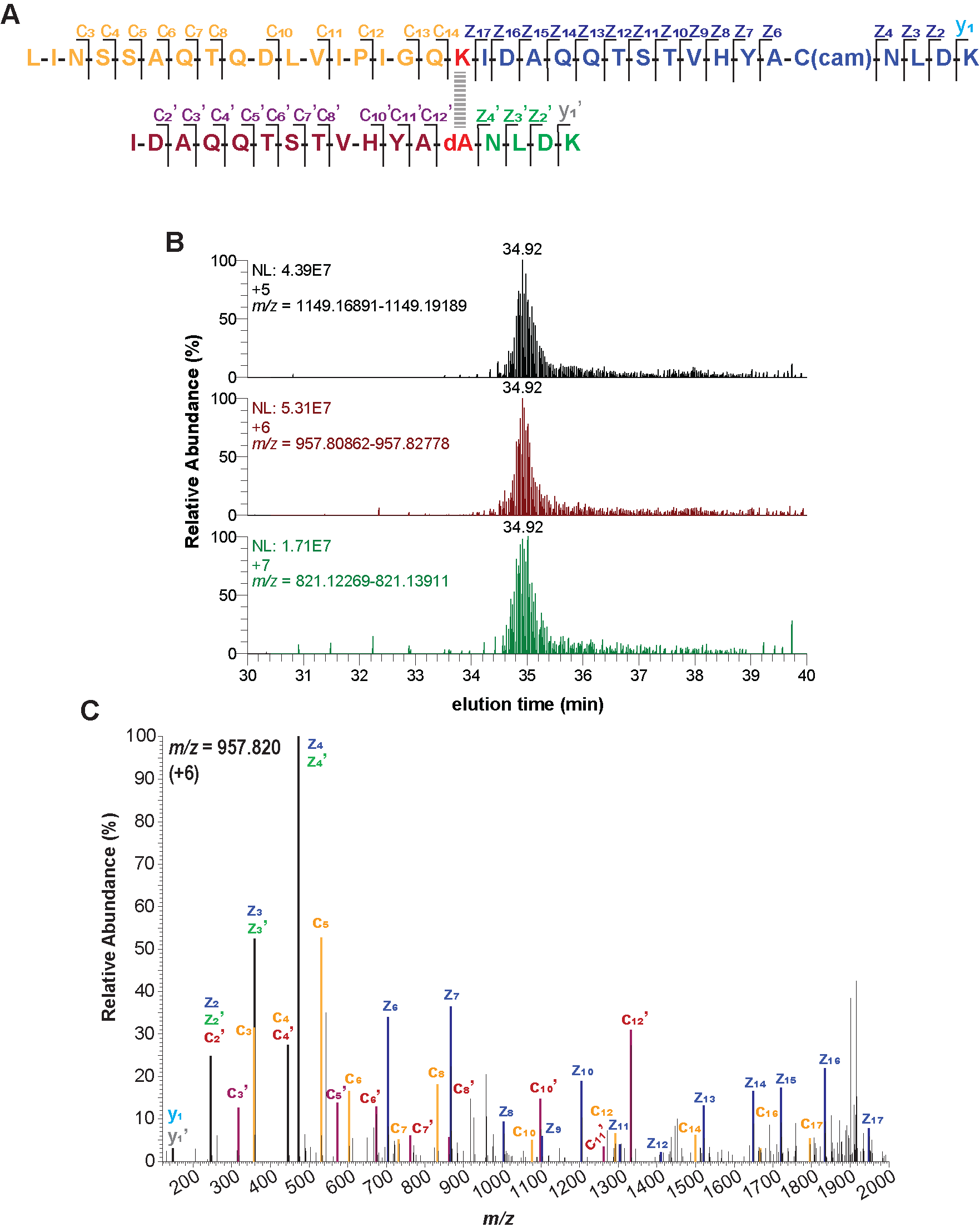


**Figure S7:** Lal-peptide detection in *T. pallidum* recombinant FlgE. (A) Trypsin-digested Lal-containing Tpa FlgE peptide with c and z ions labeled as shown in (C). Lysine-165 and DHA-178 (dA) are colored red and the Lal crosslink is represented by a dotted gray line. **(B)** XICs of the +5 (top), +6 (middle), and +7 (bottom) charged Lal crosslinked peptide shown in (A). **(C)** MS/MS ETD fragmentation spectrum of Lal-crosslinked peptide +6 parent ion with y and b ions annotated and labeled according to (A). Individual c and z ions were amplified 0-200x.


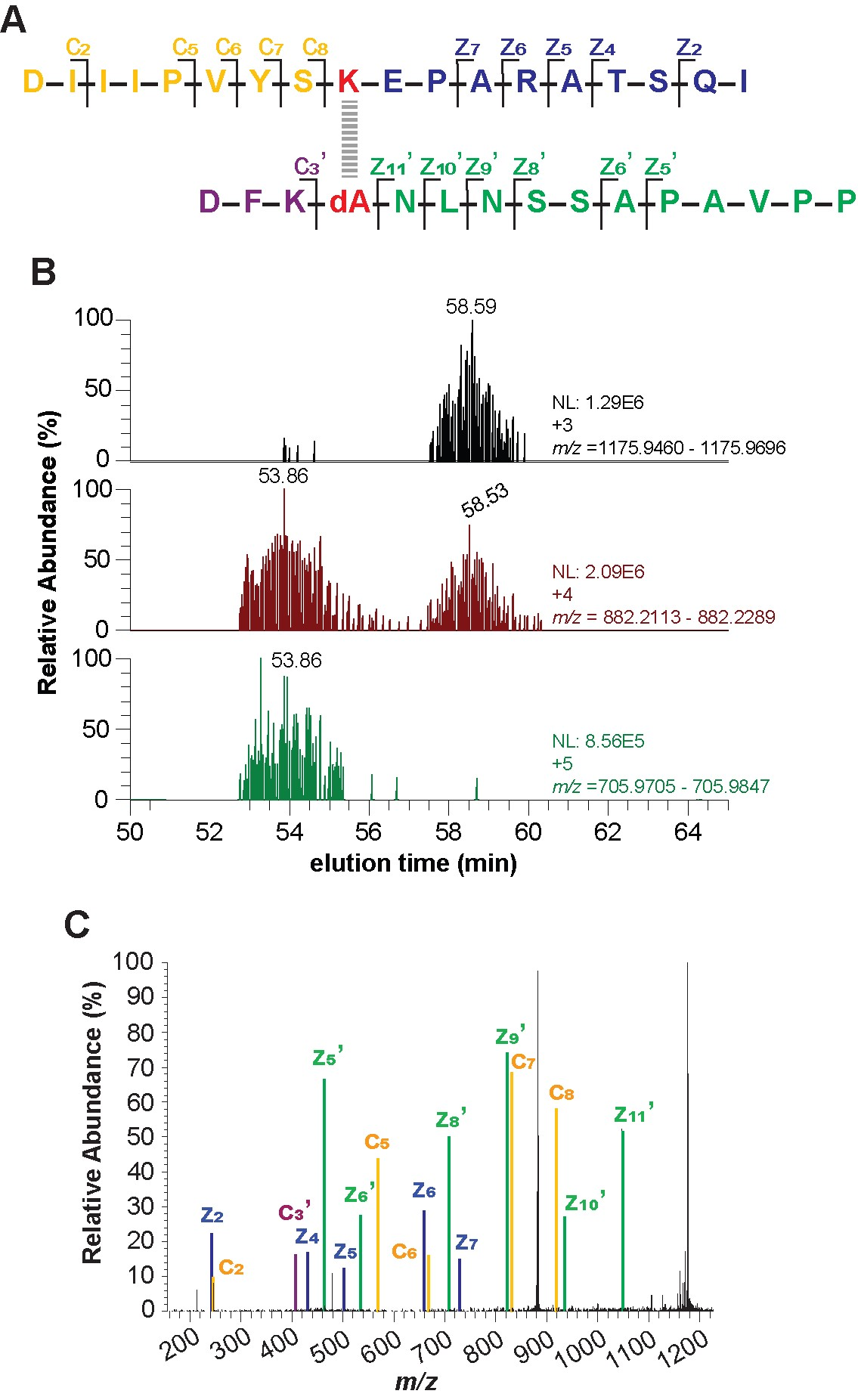


**Figure S8:** Lal-peptide detection in *L. interrogans* recombinant FlgE. (A) AspN-digested Lal-containing Li FlgE peptide with c and z ions labeled as shown in (C). Lysine-166 and DHA-179 (dA) are colored red and the Lal crosslink is represented by a dotted gray line. **(B)** XICs of the +3 (top), +4 (middle), and +5 (bottom) charged Lal crosslinked peptide shown in (A). **(C)** MS/MS ETD fragmentation spectrum of Lal-crosslinked peptide +3 parent ion with c and z ions annotated and labeled according to (A). Individual c and z ions were amplified 0-200x.


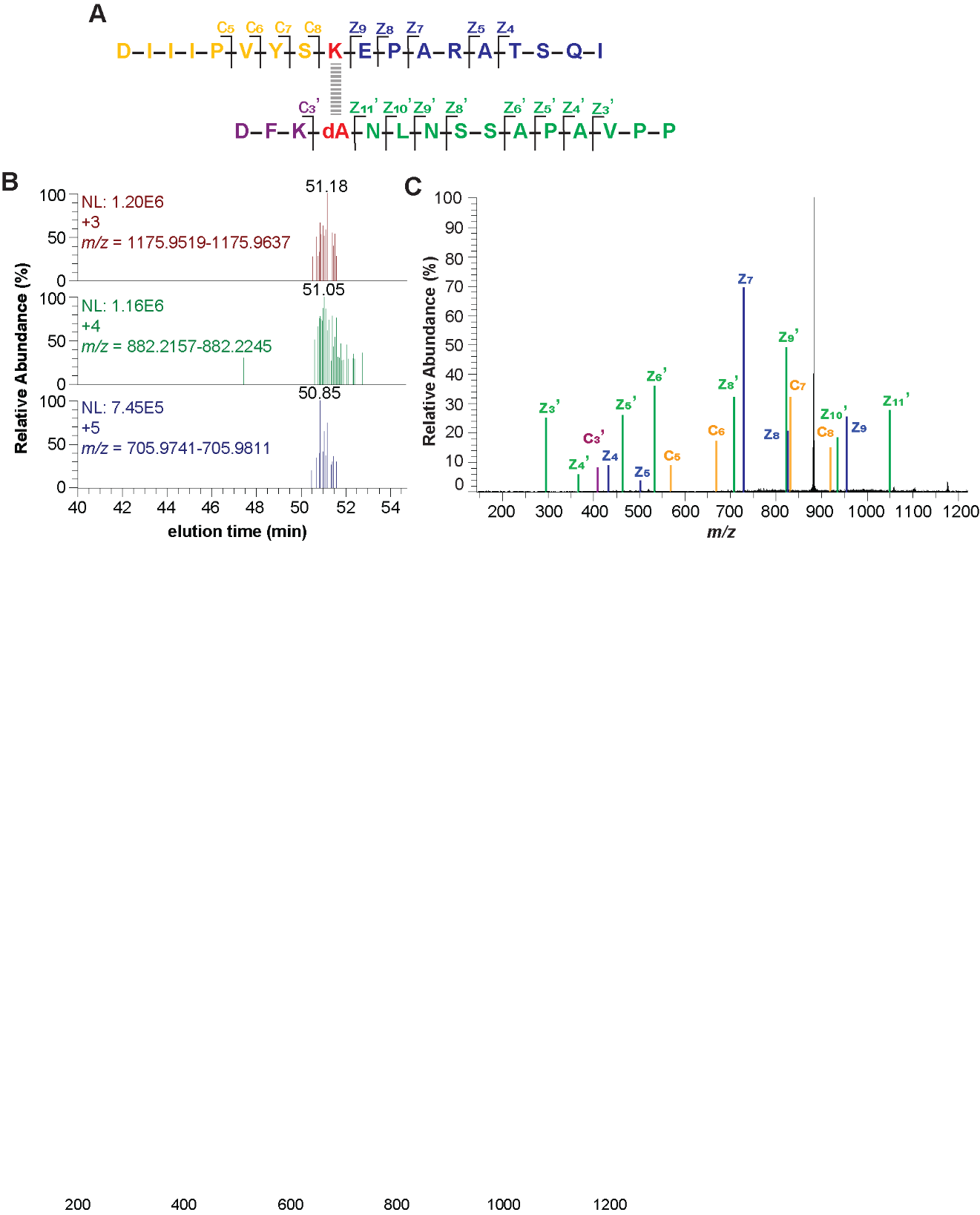


**Figure S9:** Lal-peptide detection in *L. interrogans* WT PFs. (A) AspN-digested Lal-containing Li FlgE peptide with c and z ions labeled as shown in (C). Lysine-166 and DHA-179 (dA) are colored red and the Lal crosslink is represented by a dotted gray line. **(B)** XICs of the +3 (top), +4 (middle), and +5 (bottom) charged Lal crosslinked peptide shown in (A). **(C)** MS/MS ETD fragmentation spectrum of Lal-crosslinked peptide +3 parent ion with c and z ions annotated and labeled according to (A). Individual c and z ions were amplified 0-200x.


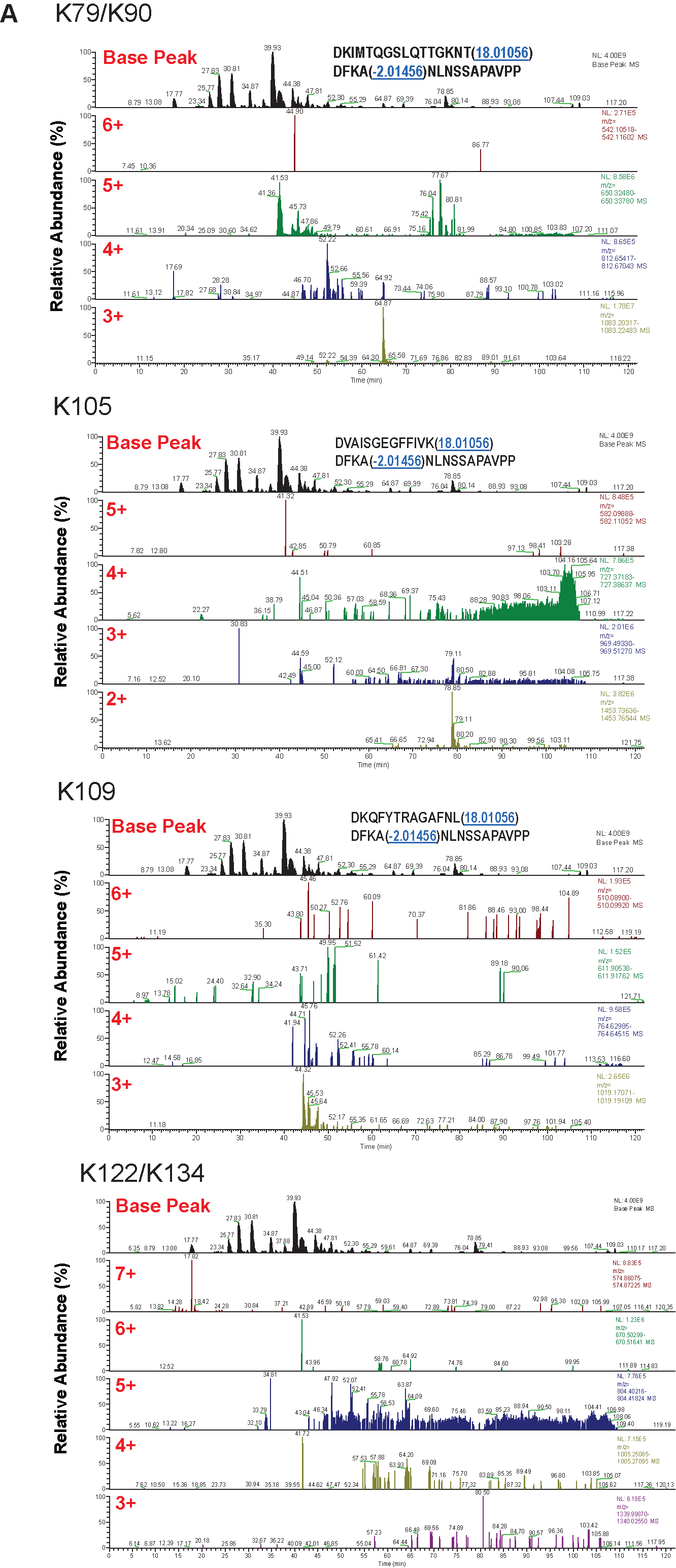


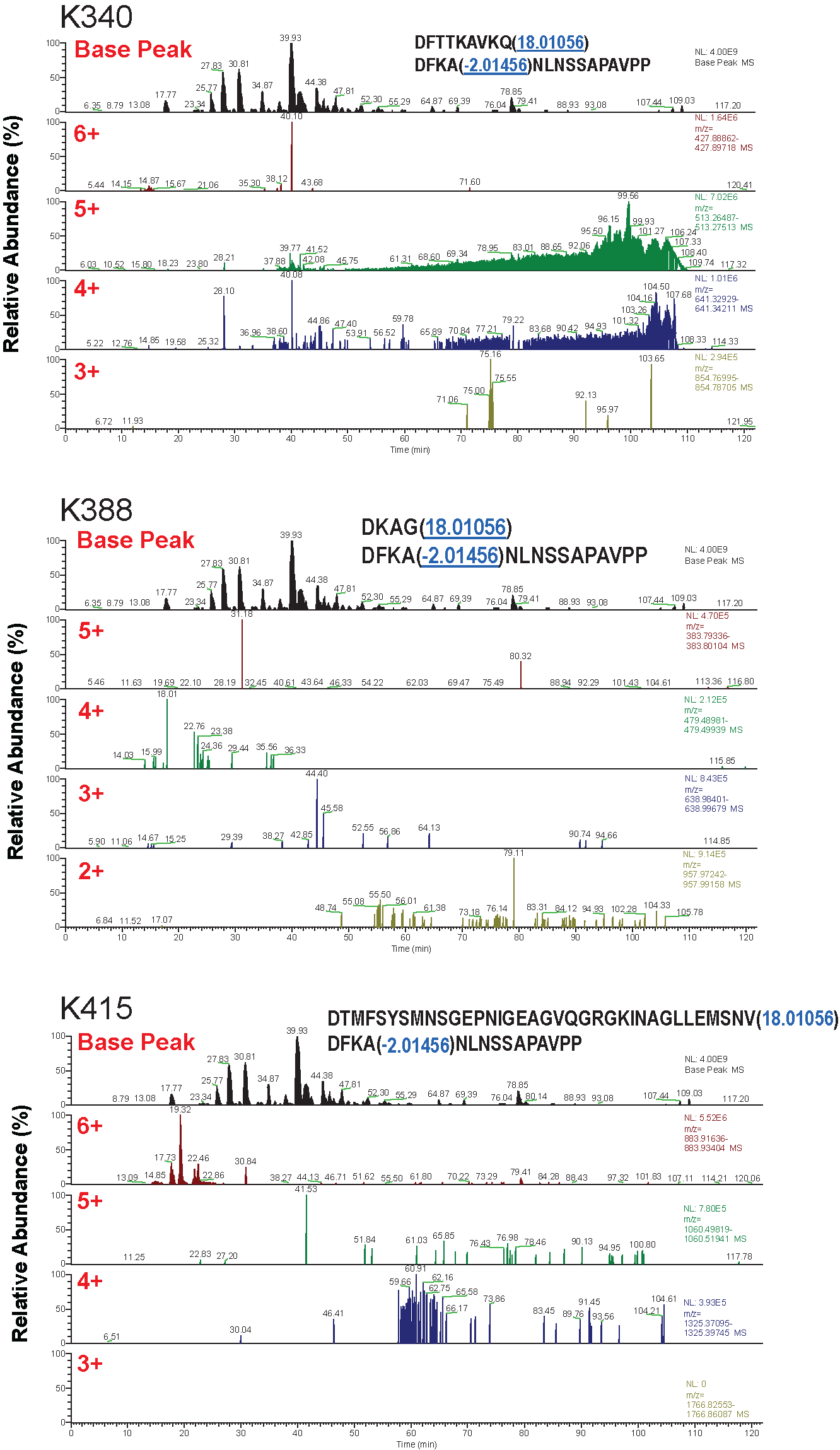


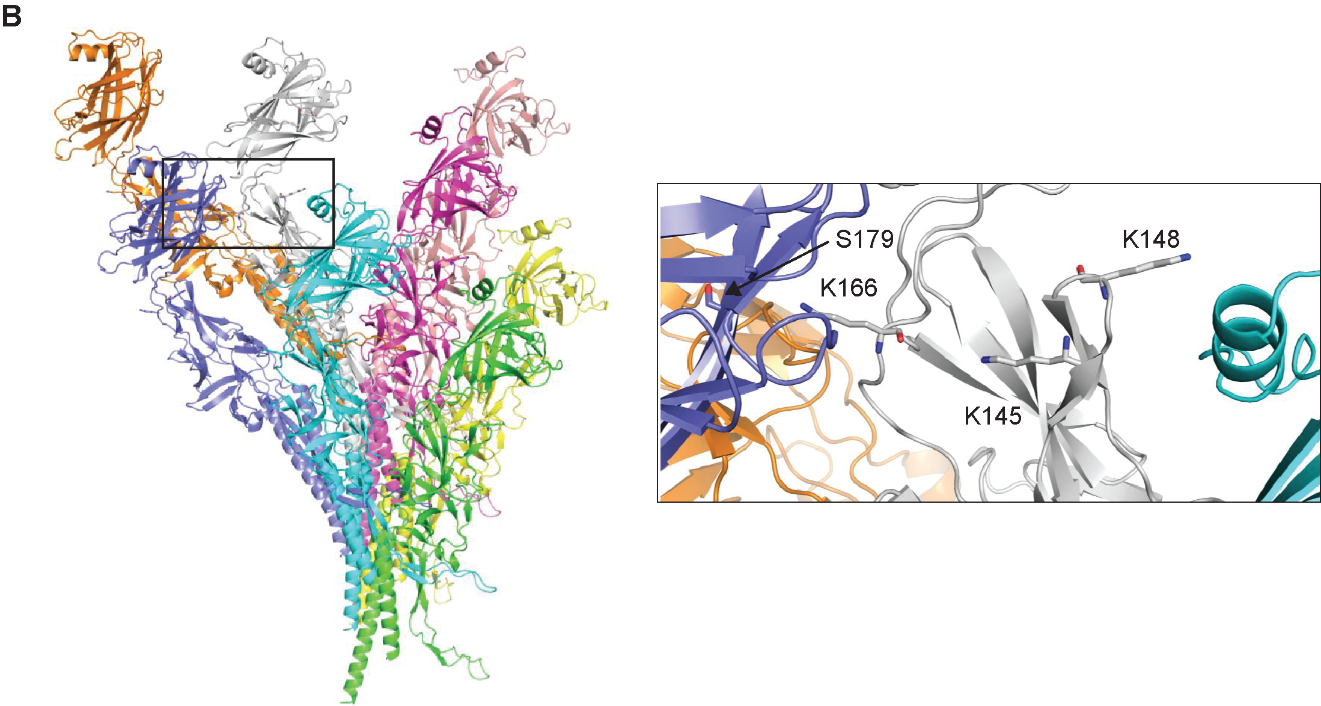


**Figure S10:** Analysis of other Lal crosslinks in *L. interrogans* FlgE. **(A)** XICs of other potential Lal crosslinking lysine residues present in Li FlgE: Lys78/90, Lys105, Lys109, Lys122/134, Lys340, Lys388, and Lys415. **(B)** Structural modeling of LiFlgE in the *Leptospira* flagellar hook. Stick models of Lys-145, Lys-148, Lys-166, and Ser-179 residues shown for comparison.


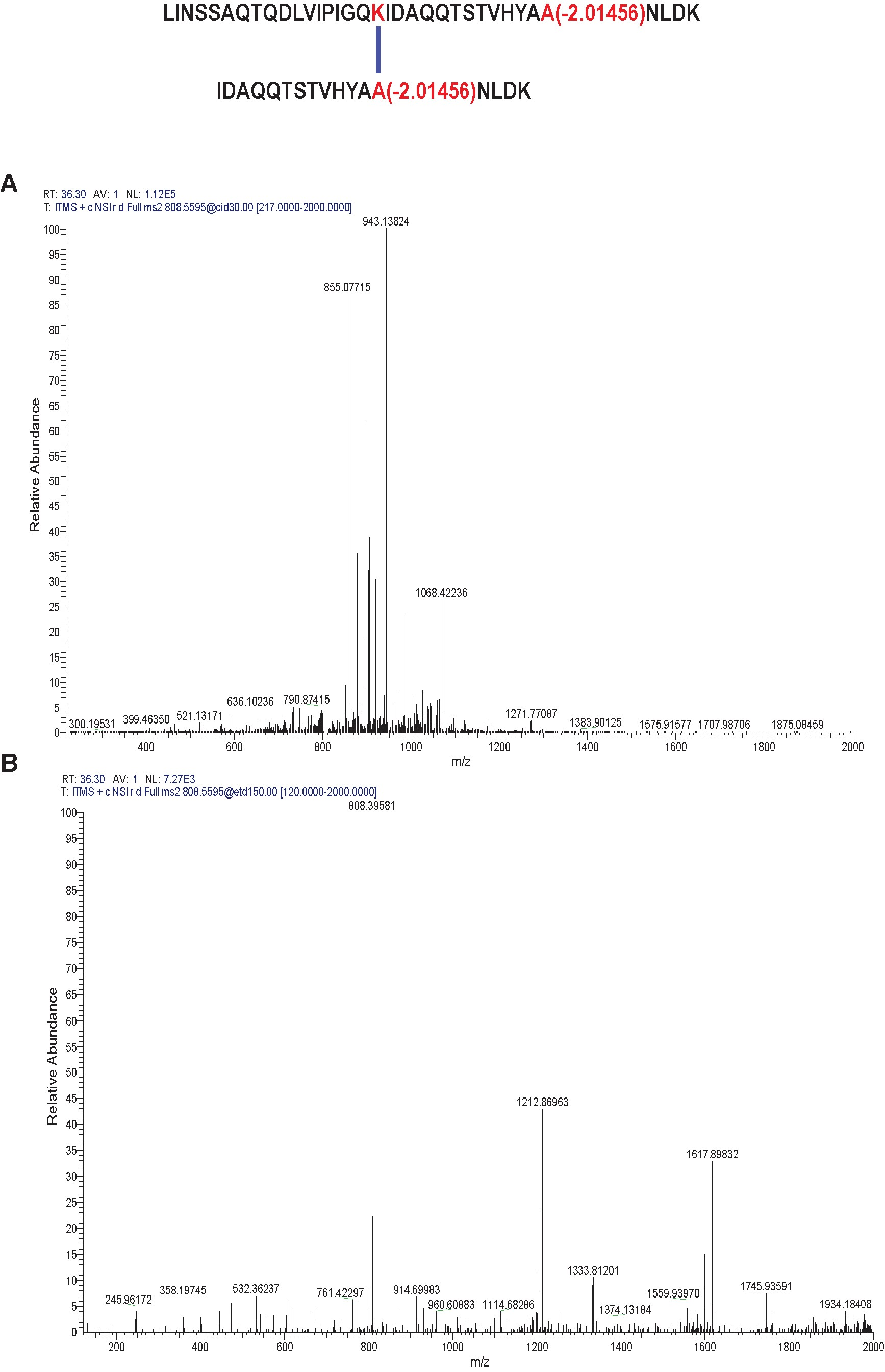


**Supplemental Figure 11:** Comparison of CID and ETD MS/MS spectra. *T. pallidum* Lal [M+7H]^7+^ crosslinked peptides (see peptide above) were fragmented using either using either **(A)** CID or **(B)** ETD methods. These data reveal that ETD produced a greater number of fragment ions at higher intensity than CID for the higher charged parent Lal-crosslinked peptides.

**Table S1:** Oligonucleotide primers used in this study

Primers Sequences(5'-3') Note ^a^

P1 AATGTTACTTTTGCTGCTAATCTTGATAAGAGA FlgE C178A mutation; [F]

P2 TCTCTTATCAAGATTAGCAGCAAAAGTAACATT FlgE C178A mutation; [R]

P3 AAATGCTTGGTGAGAGTGGT FlgE upstream;[F]

P4 ACGTTTCCCGTTGAATATGGCTCAT ATAATTATTC CTCCAAACCT FlgE upstream;[R]

P5 ATGAGCCATATTCAACGGGA *Kan* cassette; [F]

P6 TTAGAAAAACTCATCGAGCA *Kan* cassette; [R]

P7 TTTGATGCTCGATGAGTTTTTCTAA TCTAAGATTGTTTTTTTAGT FlgE downstream;[F]

P8 TCTGCACCATCAACAACAAG FlgE downstream;[R]

P9 GT AGGTTTGGAG GAATAATTAT ATGATGAGGT CTTTATATTC TG FlgE*; [F]

P10 CTTCGGCGAT CACCGCTTCC CTCAT TTAAT TTTTCAATCT TACAAG FlgE*; [R]

P11 ATGAGGGAAG CGGTGATCGC CGAAG aadA1 cassette; [F]

P12 TTATTTGCCGACTACCTTGGTGATC aadA1 cassette; [R]

P13 ATAATTATTC CTCCAAACCT FlgE* upstream; [R]

P14 T CTAAGATTGT TTTTTTAGT FlgE* downstream; [F]

a * denotes the C178A; [F] forward; [R] reverse.
